# Subtype-Specific Brain Atrophy and White Matter Alterations in Mild Cognitive Impairment

**DOI:** 10.3390/brainsci16010051

**Published:** 2025-12-29

**Authors:** Liangpeng Wei, Jiaming Lu, Xin Li, Huiquan Yang, Haoyao Wang, Zhengyang Zhu, Jiu Chen, Bing Zhang

**Affiliations:** 1 Department of Radiology, Nanjing Drum Tower Hospital, Affiliated Hospital of Medical School, Nanjing University, No. 321 Zhongshan Road, Nanjing 210008, China; glyywlp2022@163.com (L.W.);; 2 Institute of Medical Imaging and Artificial Intelligence, Nanjing University, Zhongshan Road, Nanjing 210008, China; 3 Medical Imaging Center, Nanjing Drum Tower Hospital, Affiliated Hospital of Medical School, Nanjing University, Zhongshan Road, Nanjing 210008, China; 4 State Key Laboratory of Pharmaceutical Biotechnology, Nanjing University, Nanjing 210023, China

**Keywords:** amnestic and non-amnestic mild cognitive impairment, anterior cingulate cortex, inferior fronto-occipital fasciculus, automated fiber quantification

## Abstract

**Background/Objectives**: Identifying pathological distinctions among mild cognitive impairment (MCI) subtypes is important for differentiating dementia. The purpose of this study is to investigate subtype-specific structural alterations in amnestic MCI (aMCI) and non-amnestic MCI (naMCI) and evaluate their potential as imaging biomarkers for subtype classification. **Methods**: T1 and DTI MRI data from two independent cohorts were analyzed, including a discovery dataset (58 aMCI, 35 naMCI, and 95 NC) and a replication dataset (61 aMCI, 39 naMCI, and 67 NC). Surface-based morphometry and automated fiber quantification (AFQ) were used to examine cortical thickness and white matter microstructure. Mediation models were used to explore the links between brain structure and cognitive outcomes. A logistic regression model was applied to evaluate classification performance. **Results**: The aMCI exhibited right hippocampal atrophy. In the naMCI, reduced cortical thickness was observed in the right anterior cingulate cortex (rACC) and opercular inferior frontal gyrus, along with increased fractional anisotropy (FA) in the right inferior fronto-occipital fasciculus (IFOF). These alterations were linked to domain-specific cognitive deficits. Moreover, partial mediation effects of IFOF FA values were observed in the link between rACC thickness and cognitive outcomes. Furthermore, these structural alterations effectively distinguished between aMCI and naMCI, showing stable performance across independent datasets (Accuracy = 0.821, AUC = 0.904). **Conclusions**: Our findings reveal distinct structural alterations across MCI subtypes, providing deeper insight into the heterogeneous mechanisms of dementia and supporting the potential of imaging markers for the diagnosis of MCI subtypes.

## 1. Introduction

Mild cognitive impairment (MCI) is widely regarded as a prodromal phase between healthy aging and dementia, and its amnestic (aMCI) and non-amnestic (naMCI) subtypes show distinct trajectories toward different dementias. Neuroimaging differs between subtypes: aMCI exhibits Alzheimer’s disease (AD)-related changes, including medial temporal atrophy and higher amyloid burden, whereas naMCI shows lower amyloid positivity (26.5% vs. 64.7%) and alterations more linked to non-AD pathologies, such as vascular and frontotemporal dementia [1,2,3]. Notably, naMCI shows stronger associations with biopsychosocial frailty [4]. Additionally, aMCI demonstrates a higher probability of progression to dementia [5]. Given the differences between aMCI and naMCI, identifying subtype-specific imaging features can improve diagnosis and guide targeted interventions.

White matter microstructural change and gray matter atrophy are critical for understanding the progression of MCI subtypes. The aMCI shows fractional anisotropy (FA) and mean diffusivity (MD) abnormalities in the fornix, uncinate fasciculus, and parahippocampal cingulum [6,7], as well as left hippocampal atrophy [8]. naMCI exhibits lower right inferior frontal gyrus volume, the degeneration of associated fiber bundles, and reduced white matter integrity within the superior longitudinal fasciculus [7,8]. These patterns relate to functional differences, with frontal cortex dysfunction in aMCI and subcortico-frontal network abnormalities in naMCI [9].

Diffusion tensor imaging (DTI) enables the characterization of white matter microstructural properties [10]. Automated fiber quantification (AFQ) enables the quantitative assessment of fiber tracts along their anatomical trajectories [11,12]. AFQ enables quantitative analyses of specific white matter fiber bundles, providing more precise information on tract-specific microstructural integrity [13,14]. In contrast, voxel-based analyses or tract-based spatial statistics (TBSS) typically focus on the whole-brain white matter skeleton, which may overlook localized alterations within individual fiber bundles. Moreover, AFQ automatically segments white matter tracts using a standardized pipeline, thereby reducing operator-dependent bias and making it particularly suitable for multicenter studies [15]. In disorders such as Parkinson’s disease or metabolic syndrome, AFQ enables the identification of tract-specific white matter damage across distinct disease subtypes [16]. When combined with surface-based morphometry (SBM), this approach can further delineate patterns of cortical-white matter network degeneration, thereby enhancing the ability to characterize clinical heterogeneity [17,18]. In contrast, whole-brain TBSS analyses may obscure such subtle but biologically meaningful differences [19]. Despite its complementary role to voxel-based analysis or TBSS, AFQ has seldom been employed to examine white matter alterations across aMCI and naMCI subtypes. Our previous related work was limited by single-center data, a small sample size, and incomplete diffusion metric analysis [20].

To investigate structural neuropathology comprehensively in aMCI and naMCI, we combined SBM analysis for gray matter with AFQ analysis of white matter tracts, using data from two centers to enhance the reliability and generalizability. We hypothesize that differences in brain structural changes between aMCI and naMCI are associated with differential cognitive impairments and contribute to clinical diagnosis.

## 2. Materials and Methods

### 2.1. Participants

The study used two independent cohorts to confirm the result reproducibility, with both datasets encompassing aMCI, naMCI, and normal controls (NCs). The discovery dataset consisted of 58 aMCI, 35 naMCI, and 95 NCs who enrolled from July 2021 to August 2023, at the Nanjing Drum Tower Hospital. All participants underwent brain magnetic resonance imaging (MRI) scans and a neuropsychological assessment with the Clinical Dementia Rating (CDR), Mini-Mental State Examination (MMSE), Montreal Cognitive Assessment (MoCA), Memory Index Score (MIS), Boston naming test (BNT), Stroop color and word test part three (Stroop C), and Clock Drawing Test (CDT). The replication dataset consisted of 61 aMCI, 39 naMCI, and 67 NCs who enrolled from July 2020 to December 2022, at the Nanjing Brain Hospital. Similarly, all subjects underwent whole brain MRI scans and neuropsychological assessments including the CDR, MMSE, MoCA, Stroop C, CDT, Auditory Verbal Learning Test (AVLT), symbol digit modality test (SDMT), verbal fluency test (VFT), and digit span test (DST).

According to the Petersen criteria, the aMCI met the following inclusion criteria: (1) preserved global cognitive function, defined by a Clinical Dementia Rating (CDR) score ≤ 0.5 and Mini-Mental State Examination (MMSE) scores above education-adjusted cutoffs (≥17 for illiterate individuals, ≥20 for primary school education, and ≥24 for middle school education or higher); (2) subjective memory complaints persisting for at least 3 months, either reported by the patients themselves or corroborated by informants; (3) objective memory impairment was defined as performance ≥ 1.5 standard deviations below the normative mean, operationalized as an MIS score < 7 points in dataset 1 or an AVLT score < 17 points in dataset 2, with other cognitive domains remaining intact. The naMCI met the following inclusion criteria: (1) preserved global cognitive function, consistent with the criteria applied to aMCI; (2) objective non-memory cognitive impairment, defined as deficits in at least one cognitive domain other than memory, including language (Boston Naming Test and Verbal Fluency Test), visuospatial ability (Clock Drawing Test), executive function (Stroop Color Test C), and attention (Symbol Digit Modalities Test and Digit Span Test) [8,21]. Specifically, in the discovery dataset, non-memory impairment was defined by the following cutoffs: BNT score ≤ 22, Stroop C time ≥ 109, and CDT score ≤ 9. In the replication dataset, the corresponding cutoffs were VFT score ≤ 18, Stroop C time ≥ 97, SDMT score ≤ 34, DST score ≤ 12, and CDT score ≤ 8. The NCs met the following inclusion criteria: (1) absence of memory complaint; (2) cognitive performance within the normal range; (3) a CDR score of 0.

The exclusion criteria for all participants were as follows: (1) history of psychiatric conditions (e.g., schizophrenia or bipolar disorder); (2) systemic diseases with potential cognitive impact, such as hypothyroidism or anemia; (3) major neurological disorders, including Parkinson’s disease or stroke; (4) contraindications to MRI scanning (e.g., claustrophobia or metallic implants).

### 2.2. Magnetic Resonance Imaging Acquisition

In the discovery cohort, MRI data were acquired on a 3.0 T scanner equipped with an eight-channel phased-array head coil (Achieva TX dual-source parallel RF transmission system; Philips Medical Systems, Best, The Netherlands). High-resolution T1-weighted images were obtained using the following parameters: TR = 7600 ms, TE = 3400 ms, flip angle = 8°, FOV = 256 × 256 × 192 mm^3^, and slice thickness = 1 mm. DTI data were collected using a spin-echo echo-planar imaging (EPI) sequence, with diffusion sensitization applied along 32 noncollinear directions (b = 1000 s/mm^2^) and one non-diffusion-weighted image (b = 0 s/mm^2^). The acquisition parameters were as follows: TR = 9154 ms, TE = 55 ms, FOV = 224 × 224 mm^2^, slice thickness = 2.5 mm, and voxel size = 2 × 2 × 2.5 mm^3^.

In the replication cohort, MRI data were obtained on a 3.0 T scanner (Magnetom TIM Trio; Siemens, Erlangen, Germany). High-resolution T1-weighted images were acquired with TR of 1900 ms, TE of 2480 ms, FOV of 250 × 250 mm^2^, matrix size of 256 × 256, and slice thickness of 1 mm. DTI data were collected using an interleaved echo-planar imaging (IEPI) sequence with isotropic voxels of 3.0 mm^3^. Diffusion encoding was applied along 32 directions with a b value of 1000 s/mm^2^. The corresponding acquisition parameters were TR = 6600 ms, TE = 93 ms, FOV = 240 × 240 mm^2^, and matrix size = 128 × 128.

### 2.3. Magnetic Resonance Image Preprocessing

DTI data preprocessing was performed using FSL (version 5.0; FMRIB, University of Oxford, UK). Brain extraction was carried out with the BET to remove non-brain tissue. Subsequently, eddy current- and motion-related distortions were corrected by affine registration to the b0 image. Diffusion gradient directions were then reoriented, and diffusion tensors were estimated to derive the fractional anisotropy (FA), mean diffusivity (MD), axial diffusivity (AD), and radial diffusivity (RD) maps.

Three-dimensional T1-weighted images were processed with FreeSurfer v6.0.0 (https://surfer.nmr.mgh.harvard.edu/fswiki/rel6downloads (accessed on accessed on 26 January 2025)) using the automated surface-based pipeline, including segmentation, surface reconstruction, and spatial alignment. Images were registered to the Desikan–Killiany atlas, intensity normalized, and skull stripped. Tissues were segmented into white matter, gray matter, and CSF, with manual correction of errors in Freeview. Finally, a 10 mm FWHM Gaussian smoothing was applied to enhance the signal-to-noise ratio.

### 2.4. Automated Fiber Quantification (AFQ)

Preprocessed DTI and T1-weighted images were analyzed with the MATLAB (R2024a)-based AFQ toolbox to identify 20 major white matter tracts per subject. Fiber tract identification and quantification involved four main steps: (1) whole-brain deterministic tractography was performed within a white matter mask (FA > 0.3), with tracking terminated when FA < 0.2 or the turning angle exceeded 30°; (2) major white matter tracts were segmented using an automated regions of interest (ROI) based approach guided by established anatomical protocols and refined with probabilistic tract atlases; (3) tract cores were estimated by modeling each fiber group as a three-dimensional Gaussian distribution, and outlier fibers were removed; (4) diffusion metrics were calculated along the tract core using contributions weighted by each fiber’s distance from the core and summarized as 100-node tract profiles. Quality control was primarily ensured through the fiber cleaning procedures implemented in step (3): fibers with extreme lengths (>5 SD from the group mean) were removed, and the remaining fibers were resampled to 100 equidistant nodes to assess spatial consistency. Fibers deviating more than 4 SD from the tract core defined by Mahalanobis distance, were iteratively excluded. The cleaned fiber bundles were then clipped to the central segment between the two defining ROIs to reduce partial volume effects.

### 2.5. Statistical Analysis

Group characteristics were summarized using descriptive statistics. Categorical data were analyzed with chi-square tests, while continuous variables were compared using Kruskal–Wallis tests. All statistical analyses were performed in SPSS version 23.0 (IBM Corp.).

Pointwise differences in tract profiles were evaluated for FA, MD, AD, and RD across 100 nodes along 20 fiber tracts using permutation-based testing with 1000 permutations. In each permutation, subjects were randomly reassigned to three groups, and ANOVA/post hoc tests were recalculated at each location. For each tract, the longest cluster of consecutive locations showing significance (*p* < 0.05) was extracted to generate a permutation-based null distribution. Clusters exceeding the 95th percentile of this distribution were deemed significant, thereby controlling the familywise error rate [22].

Cortical thickness measures were extracted from the “lh.aparc.a2009s.stats” and “rh.aparc.a2009s.stats” files. Group comparisons of the cortical thickness were conducted on these regional measures using general linear models (GLMs), with age, sex, and years of education included as covariates. Subcortical volumes were extracted from the “aseg.stats” file. Group comparisons of subcortical volumes were conducted on these regional measures using GLMs, with age, sex, years of education, and total intracranial volume included as covariates. For both cortical and subcortical analyses, *p*-values from the omnibus GLM effects were corrected for multiple comparisons across regions using the Benjamini–Hochberg false discovery rate (FDR) procedure (q < 0.05), and regions showing significant effects after FDR correction were further examined using post hoc pairwise comparisons with least significant difference (LSD) adjustment to characterize the direction of group differences. Post hoc statistical power analyses were performed using Gpower 3.1 for the gray matter group comparisons. Observed effect sizes were derived based on the sample size and a significance level of α = 0.05, and statistical power was estimated under the given test parameters. Partial Spearman correlations assessed associations between tract measures and cortical thickness that showed significant group differences, adjusting for age, sex, and education. Mediation analysis was conducted using structural equation modeling in R statistical software (version 4.4.3) to examine the indirect effect of gray matter on cognitive test through fiber bundle microstructure, controlling for sex, age, and education. The bootstrap sensitivity analysis with 500 replications was employed to estimate confidence intervals and assess the stability of the mediation effect.

To assess the predictive value, a binary logistic regression was trained using R to discriminate between naMCI and aMCI on the discovery dataset. Class imbalance was addressed with SMOTE (oversampling aMCI and naMCI groups to *n* = 70 each). MRI features were harmonized using ComBat (https://forlhac.shinyapps.io/Shiny_ComBat/, (accessed on 5 March 2025)), with age, sex, and years of education included as covariates. Supplementary Figure S3 shows the distribution of MRI features before and after ComBat harmonization. Then a multivariable logistic regression model was constructed by jointly incorporating MRI features and demographic variables, through the linear predictor:

linear predictor = β_0_ + β_1_·IFOF_R_FA_55–64_ + β_2_·rACC thickness + β_3_·rIFGop thickness + β_4_·right hippocampal volume + β_5_·age + β_6_·sex + β_7_·years of education

Model coefficients (β_0_–β_7_) were estimated using the discovery dataset and fixed thereafter. The trained model was then applied to the independent replication dataset to generate an integrated probability score for each subject. By systematically varying the classification cutoff applied to these probability scores, the sensitivity and specificity were calculated across a range of thresholds and used to construct the ROC curve. Moreover, a five-fold cross-validation procedure within the discovery dataset was used to determine the optimal classification threshold by maximizing the Youden index [23], with the final threshold defined as the median of the fold-specific optima. The external validation confusion matrices and related performance metrics were calculated using the optimal classification threshold derived from the discovery dataset. In addition, ROC curves derived from the five-fold cross-validation on the discovery dataset were generated to assess the stability of model performance across folds and to evaluate the potential risk of overfitting.

## 3. Results

### 3.1. Demographic Characteristics and Neuropsychological Testing

Table 1 summarizes the demographic and neuropsychological data of aMCI, naMCI, and NC groups. In the discovery dataset, aMCI was older and less educated than NC, and both MCI subtypes had lower MMSE and MoCA scores than NC. The naMCI performed better than aMCI on memory tasks but worse than NC on BNT, Stroop C, and CDT. In the replication dataset, aMCI scored lower on MMSE and MoCA, with naMCI only on MoCA. In addition, naMCI showed poorer performance on the VFT, Stroop C, SDMT, and CDT, while aMCI showed more pronounced impairments in memory function.

### 3.2. Differences in White Matter Tract Profiles Among aMCI, naMCI, and NC Groups

**Figure 1:**
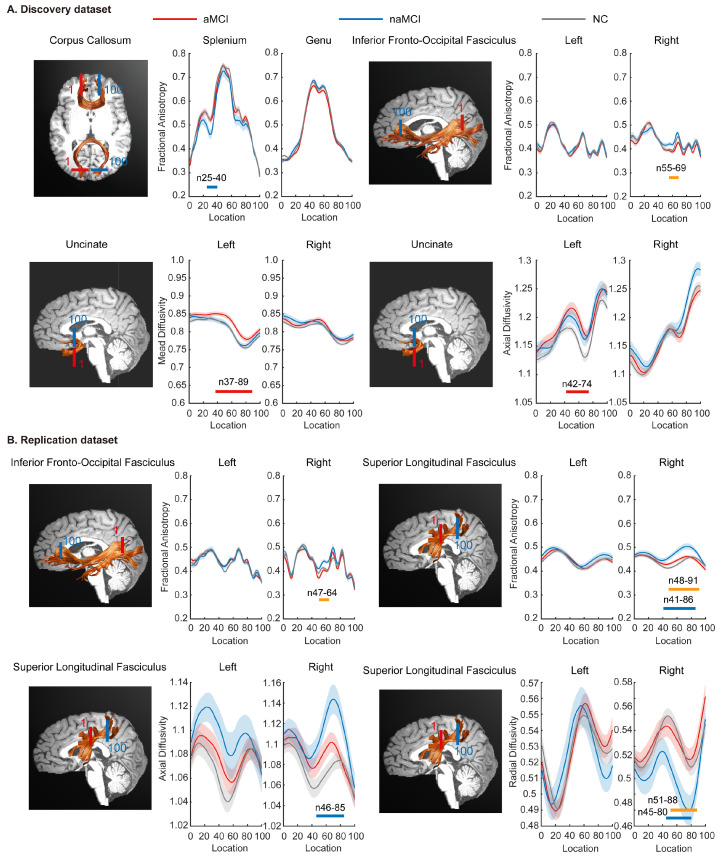
Pointwise comparisons of tract profiles across the aMCI, naMCI, and NC groups. Mean profiles (solid lines) with standard deviations (shaded areas) are shown for aMCI (red), naMCI (blue), and NC (gray) in the discovery dataset (**A**) and the replication dataset (**B**). Color bars beneath the profiles denote regions with significant between-group differences: red indicates aMCI versus NC, blue indicates naMCI versus NC, and yellow indicates aMCI versus naMCI. The *x*-axis corresponds to normalized positions along each tract, spanning from the start to the end waypoint regions of interest.

In the discovery dataset, naMCI showed reduced FA in the left splenium of the CC compared to NC and increased FA in the intermediate right IFOF (IFOF_R_FA_55–69_) compared to aMCI. In addition, aMCI exhibited increased MD (UF_L_MD_37–89_) and AD (UF_L_AD_42–74_) at the frontier and intermediate component of the left UF compared to NC. In the replication dataset, naMCI also showed significant FA (IFOF_R_FA_47–64_) increase in the intermediate right IFOF compared to aMCI. Additionally, naMCI displayed elevated FA (SLF_R_FA_41–91_) and AD (SLF_R_AD_46–85_), coupled with reduced RD (SLF_R_RD_45–88_), in the intermediate and posterior part of the right SLF. The number and position of points with significant difference in tract profiles are described in Supplementary Table S1, with corresponding fiber tracts and profiles illustrated in Figure 1. In addition, we reanalyzed the DTI data with more stringent fiber cleaning settings by reducing the deviation threshold from the tract core (from 4 SD to 3.5 SD) and narrowing the acceptable fiber length range relative to the mean length (from 5 SD to 4 SD), to further minimize potential tract contamination. Intergroup comparisons of the IFOF still revealed significant differences in the local FA values of the right IFOF for naMCI across both datasets (Supplementary Figure S1).

### 3.3. Differences in Gray Matter Among the Three Groups

For cerebral cortex, participants with naMCI consistently demonstrated cortical atrophy in the right anterior cingulate (rACC; FreeSurfer label: rh_G_and_S_cingul_Ant) and the right opercular part of inferior frontal gyrus (rIFGop; FreeSurfer label: rh_G_front_inf_Opercular) across both datasets (Supplementary Figure S2). In the discovery dataset, additional atrophic regions in naMCI included the right orbital gyrus, superior temporal gyrus, orbital sulcus, and suborbital sulcus. In the replication dataset, additional atrophy of the right middle frontal gyrus, superior frontal gyrus, and angular gyrus is observed in naMCI. The detailed difference between the three groups is shown in Table 2. Regarding the subcortical nuclei, aMCI showed atrophy mainly in the right hippocampus compared to naMCI and NC in both datasets (Table 3).

### 3.4. Correlation Between Structural Brain Alterations and Neuropsychological Scores

**Figure 2:**
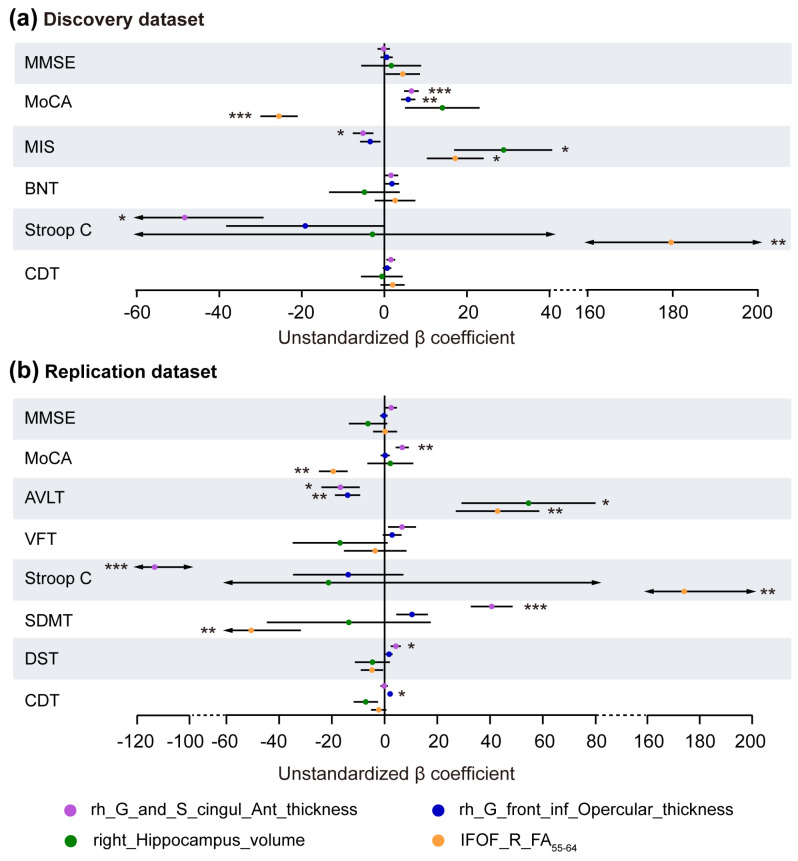
Regression analysis between brain alterations and neuropsychological test scores in MCI. Forest plots show the results in the discovery dataset (**a**) and the replication dataset (**b**). The *X* axis shows the unstandardized β. *p* values were adjusted for age, sex, and years of education. MMSE, Mini Mental State Examination; MoCA, Montreal Cognitive Assessment; MIS, Memory Index Score; BNT, Boston naming test; Stroop C, Stroop Word Color Test C; CDT, Clock Drawing Test; AVLT, Auditory Verbal Learning Test; VFT, verbal fluency test; SDMT, symbol digit modality test; DST, digit span test; rh, right hemisphere; G_and_S_cingul_Ant, anterior cingulate gyrus and sulcus; G_front_inf_Opercular, inferior frontal gyrus, opercular part; IFOF, inferior fronto-occipital fasciculus; FA, fractional anisotropy. * *p* < 0.05, ** *p* < 0.01, *** *p* < 0.001.

In MCI patients (combining aMCI and naMCI) across both datasets, the thickness of the rACC exhibited a significant positive correlation with the MoCA scores and a negative correlation with the time of Stroop C. In contrast, the IFOF_R_FA_55–64_ showed the inverse results. Further, the thickness of the rACC was negatively associated with memory performance, whereas the IFOF_R_FA_55–64_ and right hippocampus volume showed positive associations with the scores of memory domain. Details for other cognitive domains are provided in Figure 2. The corresponding regression model results, including the effect size and confidence intervals, are shown in Supplementary Table S2.

### 3.5. Relationship Between White Matter Microstructure and Cortical Thickness

**Figure 3:**
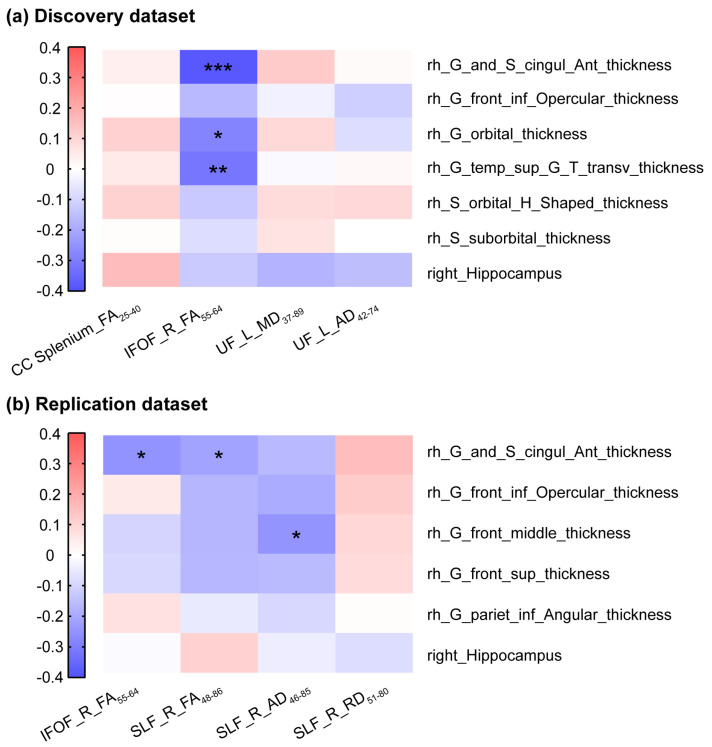
Correlation between white matter microstructure and cortical thickness. Correlation matrix showing Spearman correlation coefficients between diffusion parameters of fiber tracts and cortical thickness in discovery dataset (**a**) and replication dataset (**b**). The color of each square designates the value of the correlation coefficient: red designates a strong positive correlation; blue designates a strong negative correlation; and white designates no correlation. Asterisks designate the *p* value. G_and_S_cingul_Ant, anterior cingulate gyrus and sulcus; G_front_inf_Opercular, inferior frontal gyrus, opercular part; G_orbital, orbital gyrus; G_temp_sup_G_T_transv, superior temporal gyrus, transverse temporal gyrus; S_orbital_H_Shaped, orbital sulcus, H-shaped; S_suborbital, suborbital sulcus; G_front_middle, middle frontal gyrus; G_front_sup, superior frontal gyrus; G_pariet_inf_Angular, inferior parietal lobule, angular gyrus; rh, right hemisphere; CC, corpus callosum; IFOF, inferior fronto-occipital fasciculus; UF, uncinated fasciculus; SLF, superior longitudinal fasciculus; L, left; R, right; FA, fractional anisotropy; MD, mean diffusivity; AD, axial diffusivity; RD, radial diffusivity. All coefficients were calculated using partial correlation analysis, controlling for age, sex, and education, * *p* < 0.05, ** *p* < 0.01, *** *p* < 0.001.

In the discovery dataset, the IFOF_R_FA_55–64_ showed significant negative correlations with the cortical thickness of the rACC (r = −0.385, *p* < 0.001), right orbital gyrus (r = −0.283 *p* = 0.010), and superior–transverse temporal gyrus (r = −0.316 *p* = 0.004) in MCI participants (Figure 3a). In the replication dataset, the thickness of the rACC was associated with the IFOF_R_FA_55–64_ (r = −0.249, *p* = 0.022) and the SLF_R_FA_48–86_ (r = −0.216, *p* = 0.047) (Figure 3b). Moreover, the thickness of right middle frontal gyrus correlated with the SLF_R_AD_46–85_ (r = −0.246, *p* = 0.023).

### 3.6. Mediation Analyses

**Figure 4:**
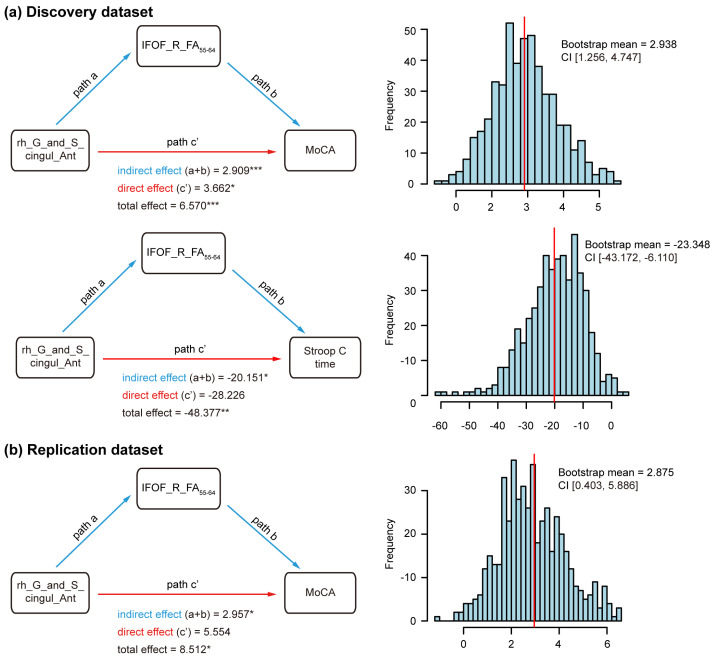
Mediation analysis. Path models illustrate the mediating role of fiber bundle characteristics in the relationship between cortical thickness and cognitive test scores in the discovery (**a**) and replication (**b**) datasets. Bootstrap sensitivity analysis shows the distribution of the indirect effect from 500 bootstrap replications, with the mean estimate and 95% confidence interval, while the red line indicates the original mediation effect. MoCA, Montreal Cognitive Assessment; Stroop C, Stroop Word Color Test C; G_and_S_cingul_Ant, anterior cingulate gyrus and sulcus; IFOF, inferior fronto-occipital fasciculus; R, right; FA, fractional anisotropy; CI, 95% confidence interval. Path model tests were performed controlling for age, sex, and years of education, * *p* < 0.05, ** *p* < 0.01, *** *p* < 0.001.

In the discovery dataset, we found that the IFOF_R_FA_55–64_ exhibited a mediation effect on the relationship between the cortical thicknesses of the rACC with MoCA scores, as well as the Stroop C times in MCI subjects. For the mediation analysis involving MoCA scores, the sensitivity analysis based on 500 bootstrap resamples yielded a mediation estimate of 2.938 (95% CI: 1.256 to 4.747), which was comparable to the original estimate of 2.909. Similarly, the bootstrap-based mediation estimate for Stroop C times was comparable to the original estimate (Figure 4a). Meanwhile, the same mediation effect of the IFOF_R_FA_55–64_ was observed on the relationship between the rACC thicknesses with MoCA scores in the replication dataset, supported by sensitivity analysis (mediation estimate = 2.875, 95% CI: 0.403–5.886) (Figure 4b). However, the IFOF FA value did not exhibit a comparable mediation pattern in the association between rACC thickness and Stroop C times.

### 3.7. Validation of Structural Abnormalities for Classification Between aMCI and naMCI

**Figure 5:**
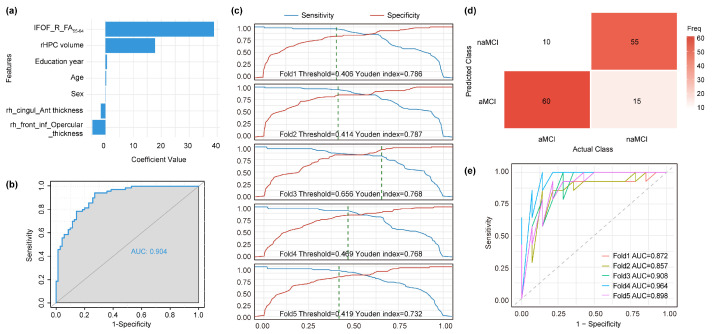
Classification of naMCI and aMCI. (**a**) The logistic regression coefficients were calculated to quantify the contribution of each feature to the diagnosis of naMCI. (**b**) ROC curves for identifying naMCI from MCI patients through external validation. (**c**) Threshold-dependent sensitivity (blue) and specificity (red) curves for all five cross-validation folds, where vertical dashed lines mark the probability thresholds that optimize the Youden index. (**d**) Confusion matrices for single trial classification of naMCI and aMCI using replication data. The color in each cell depicts the number of actual subgroup individuals predicted within each subgroup category. (**e**) ROC curves of the five-fold cross validation for distinguishing naMCI from MCI in the discovery dataset. IFOF, inferior fronto-occipital fasciculus; FA, fractional anisotropy; rHPC, Right_Hippocampus; rh, right hemisphere; cingul_Ant, anterior cingulate; front_inf_Opercular, inferior frontal gyrus, opercular part.

The simple logistic regression model used the cortical thicknesses of the rACC and the rIFGop, right hippocampus volume, IFOF_R_FA_55–64_, age, education years, and sex as features. Among all features, the right IFOF FA value emerged as the most important predictor for classification. In particular, individuals with higher IFOF FA values were more likely to be diagnosed with naMCI (Figure 5a). When externally validated using the replication dataset, the model achieved robust performance with an AUC of 0.904 (Figure 5b). The optimal probability threshold was confirmed using five-fold cross-validation on the discovery dataset based on the Youden index, and the final threshold of 0.419 was set as the median of the five optimal values (Figure 5c). External validation showed relatively good performance (accuracy 0.821, specificity 0.857, sensitivity 0.786, precision 0.786) (Figure 5d). In addition, internal five-fold cross-validation showed stable model performance (Figure 5e). The corresponding internal validation classification results and performance metrics are shown in Supplementary Figure S4.

## 4. Discussion

This study aimed to characterize differential structural alteration patterns between aMCI and naMCI and to evaluate their diagnostic relevance. In two independent cohorts, naMCI exhibited reduced cortical thickness in the right ACC and IFGop, together with increased FA in the middle segment of the right IFOF. In addition, the IFOF FA values played a significant mediation role in the relationship between the right ACC thickness and MoCA scores. These alterations enabled subtype classification with 82% accuracy (AUC = 0.904).

Our results revealed reduced cortical thickness in the rACC and rIFG in naMCI subjects. Previous meta-analyses reported the atrophy of the ACC exclusively in naMCI [1,24], while evidence regarding the involvement of the IFG has been more variable. Some studies observed IFG atrophy in both aMCI and naMCI [1], whereas others found the volume reduction in the right IFG in naMCI specifically [8], which supports our current results. The ACC is involved in error detection, attention, motivation, and emotional regulation [25,26,27]. Notably, its role in error detection has been well documented through the Stroop task [28,29]. Consistent with this, our data revealed a positive correlation between the right ACC and the Stroop task performance. In addition, the ACC thickness was associated with better performance in SDMT and DST in replication dataset. It is reported that enhanced ACC structural connectivity and activation improve SDMT [30] and DST [31] scores, underscoring the involvement of the ACC in executive function and attention. This selective atrophy may be related to degenerative processes in non-memory domains such as tau protein deposition or vascular lesions [32,33,34], consistent with the clinical progression of naMCI toward frontotemporal degeneration or vascular dementia [35]. By contrast, individuals with aMCI exhibited pronounced atrophy of the right hippocampus. The hippocampus plays a central role in episodic memory encoding and consolidation and is especially susceptible to degeneration in aMCI [36,37]. Longitudinal neuroimaging research has demonstrated that right hippocampal atrophy strongly predicts conversion from aMCI to AD dementia [38]. Collectively, the distinct structural alteration patterns observed in aMCI and naMCI may reflect divergent pathological mechanisms underlying their progression toward different dementia.

Regarding white matter, naMCI patients showed increased IFOF_R_FA_55–64_ in both datasets, while the SLF_R_FA_41–91_ and SLF_R_AD_46–85_ were significantly increased in the replication dataset. In WMH-MCI, the diffusion metrics of the IFOF and SLF appear particularly vulnerable to vascular pathology, with the MD values in the anterior segment of the right IFOF showing a negative correlation with the MMSE scores [39]. In idiopathic Parkinson’s disease–related MCI, increased FA in the anterior segment of the right IFOF was positively associated with MoCA performance and executive function [40]. In our study, the middle right IFOF FA was negatively associated with MoCA and positively with memory scores, reflecting functional heterogeneity along the IFOF. Specifically, the anterior segment is more involved in executive functions, and the middle segment is related to memory domain. The observed FA elevations of IFOF in naMCI possibly indicate a compensation for structural reorganization to temporarily support memory function [41]. Elevated FA is generally interpreted as reflecting enhanced microstructural organization, potentially related to increased axonal density, improved myelination, or higher fiber coherence. However, increased FA may indicate compensatory neural reorganization in certain pathological contexts. For example, patients with aphasia have shown elevated FA in the left SLF and UF, possibly reflecting compensatory remodeling of language networks [13]. Similarly, FA values in schizophrenia spectrum disorders often fall between those of healthy controls and patients with schizophrenia, suggesting partial compensatory white matter reorganization [42]. Notably, excessive regional compensation may come at the expense of global network efficiency. For instance, overactivation of the IFOF could disrupt the dynamic balance between the default mode network and attentional networks, thereby reducing the overall cognitive integration [43,44]. This abnormal increase in FA is unlikely to be attributable to CSVD or frontotemporal dementia, as FA abnormalities in these conditions are typically localized to deep white matter regions or subcortical pathways. In CSVD, elevated FA has been reported in deep gray matter nuclei such as the thalamus, where it is thought to reflect microstructural alterations, including cytotoxic edema or changes in water compartmentalization, rather than preserved white matter integrity [45]. FA alterations in AD-related MCI primarily involve the pathway connecting the basal forebrain and the entorhinal cortex, while frontotemporal lobar degeneration exhibits widespread white matter abnormalities in the frontal lobe [46,47]. In addition, increased FA may also reflect reactive gliosis or inflammatory responses following neural injury, resulting in restricted radial diffusion and higher anisotropy without implying preserved or improved tissue integrity, as reported in studies of mild traumatic brain injury and Parkinson’s disease [48,49]. Therefore, the increased FA observed in the present study should be interpreted with caution and requires further investigation.

In both datasets, the IFOF_R_FA_55–64_ was negatively correlated with the rACC thickness and mediated its relationship with MoCA scores. Despite lacking direct anatomical connections, the IFOF and ACC are functionally coupled. The IFOF connects the frontal, parietal, and occipital lobes to integrate visual information with higher-order cognitive processes [40,50], while the ACC dynamically interacts with executive and default mode networks via IFOF [51]. Pathological co-alterations in these regions may disrupt frontal–limbic–occipital circuits, contributing to cognitive, emotional, and motor dysfunction [52,53,54]. The mediation effect of the IFOF may reflect the imbalance of the default mode network (DMN), given the excessive compensation of the IFOF described above [55]. Indeed, the abnormal functional connectivity of the DMN and executive control network (ECN) is a key characteristic of naMCI. Previous studies have reported increased connectivity within the DMN in patients with naMCI [56]. In addition, enhanced connectivity has been observed in the prefrontal–parietal control network [57]. Notably, stronger coupling between the DMN and the frontoparietal network may indicate abnormal functional integration between the frontal and posterior brain regions, including the occipital lobe [58]. Furthermore, the mediating role of IFOF_R_FA_55–64_ in the relationship between rACC thickness and executive performance was evident in the discovery dataset but not confirmed in the replication dataset. The differences in mediation patterns observed between datasets may reflect not only participant heterogeneity, given that individuals with MCI show impairments across multiple cognitive domains, but also methodological differences arising from non-uniform cognitive assessment protocols. Future studies could conduct multicenter validation of the mediation effect of the IFOF in MCI patients characterized by impairment in a specific cognitive domain. Furthermore, in the discovery dataset, the local FA values of the retained IFOF continued to show a negative correlation with the rACC thickness when using stricter clarity criteria, whereas in the replication dataset, the reduced sample size resulting from the failure to retain the IFOF in some patients may have contributed to the loss of statistical significance (Supplementary Figure S1).

Finally, external validation confirmed that the identified structural brain differences performed well in distinguishing between aMCI and naMCI (accuracy = 82%). Few studies have focused on differentiating the two subtypes using imaging features. A previous study demonstrates that machine learning models using written picture description tasks can accurately distinguish between amnestic and non-amnestic MCI, achieving up to 90% classification accuracy [59]. However, the process requires active patient participation, which may pose practical limitations in clinical diagnosis. Furthermore, gait features enabled highly accurate classification between aMCI and naMCI (accuracy > 95%) [60], but the lack of validation on an independent external dataset limits the generalizability of the findings. In summary, the imaging biomarkers identified in our study are independent of behavioral cognitive tests and may serve as complementary objective references alongside cognitive scales, with potential clinical relevance for the differential diagnosis of amnestic and non-amnestic MCI.

This study also has several limitations as follows. First, the naMCI groups included only 35 subjects in the discovery dataset and 39 subjects in the replication dataset. To assess the reliability of results, we calculated the statistical power (the discovery dataset: cortical thickness of the rACC, 0.97; thickness of the rIFGop, 0.98; the replication dataset: thickness of the rACC, 0.78; thickness of the rIFGop, 0.97). These results indicate that the statistical power was generally adequate. Although consistent rACC atrophy was observed across both cohorts, the modest statistical power in the replication dataset limits sensitivity to subtle effects. Therefore, the replication results should be interpreted cautiously, and larger independent samples will be required to strengthen confidence in rACC atrophy in naMCI. Second, although standard preprocessing and quality control were applied, quantitative head motion metrics were not included, limiting direct assessment of motion effects on diffusion measures, particularly in the MCI group. Future studies with larger sample sizes and optimized acquisition protocols should incorporate quantitative motion parameters to better control for motion-related bias. Third, tract segmentation using alternative toolboxes such as diffusion imaging in python (DIPY) may further enhance segmentation accuracy, as DIPY incorporate more advanced modeling strategies, including probabilistic tractography and learning-based segmentation, which may improve bundle delineation. Although additional analyses with stricter fiber cleaning supported the stability of our findings, future studies using multiple tract segmentation pipelines and cross-method validation are needed to further strengthen confidence in white matter tract-based results.

## 5. Conclusions

This two-center study demonstrates that naMCI is characterized by atrophy of the right ACC accompanied by increased FA values of right IFOF, both of which are associated with impairments in distinct cognitive domains. Furthermore, the IFOF FA values play a mediation role in the relationship between rACC thickness and cognition. These structural abnormalities appear to be promising neuroimaging indicators for differentiating MCI subtypes.

## Figures and Tables

**Table 1:** Demographics of participants.

	aMCI	naMCI	NC	*p* Value	Post Hoc Groupwise		
					aMCI vs. naMCI	aMCI vs. NC	naMCI vs. NC
Discovery dataset							
Number of subjects	58	35	95				
Education (years)	11.10 ± 3.36	13.00 ± 4.29	12.73 ± 4.04	0.009	0.126	0.008	1.000
Age (year)	67.33 ± 8.02	61.73 ± 8.35	62.18 ± 8.15	<0.001	0.008	0.001	1.000
Sex (M/F)	31/27	14/21	53/42	0.148	–	–	–
MMSE	27.53 ± 1.66	27.00 ± 1.92	29.06 ± 0.95	<0.001	0.835	<0.001	<0.001
MoCA	21.40 ± 2.37	19.85 ± 1.64	27.66 ± 1.26	<0.001	0.214	<0.001	<0.001
MIS	2.66 ± 2.01	6.94 ± 3.59	12.32 ± 2.24	<0.001	0.001	<0.001	<0.001
BNT	21.67 ± 1.70	20.67 ± 2.53	22.34 ± 1.77	0.005	0.092	0.026	<0.001
Stroop C (second)	97.02 ± 19.39	114.03 ± 27.03	82.07 ± 15.00	<0.001	0.022	<0.001	<0.001
CDT	8.79 ± 1.17	8.48 ± 1.37	9.52 ± 0.62	<0.001	1.000	0.002	<0.001
Replication dataset							
Number of subjects	61	39	67				
Education (years)	11.87 ± 2.74	12.62 ± 2.96	12.22 ± 2.55	0.421	–	–	–
Age (year)	65.34 ± 7.29	64.64 ± 8.21	62.85 ± 7.48	0.189	–	–	–
Sex (M/F)	28/36	17/22	23/44	0.477	–	–	–
MMSE	26.90 ± 1.97	27.92 ± 2.02	28.57 ± 1.28	<0.001	0.008	<0.001	0.479
MoCA	22.13 ± 2.34	22.56 ± 2.57	26.19 ± 1.49	<0.001	1.000	<0.001	<0.001
AVLT	17.16 ± 1.70	27.38 ± 7.79	37.40 ± 3.27	<0.001	<0.001	<0.001	0.002
VFT	18.41 ± 4.62	16.77 ± 5.71	20.34 ± 4.58	0.004	0.906	0.049	0.005
Stroop C (second)	84.48 ± 24.03	100.59 ± 34.78	79.87 ± 16.40	0.008	0.134	0.659	0.006
SDMT	33.74 ± 9.40	29.38 ± 7.34	39.25 ± 9.39	<0.001	0.069	0.005	<0.001
DST	11.23 ± 2.08	11.46 ± 1.34	12.64 ± 1.91	<0.001	1.000	0.001	0.007
CDT	8.74 ± 1.26	8.18 ± 1.28	9.37 ± 0.95	<0.001	0.099	0.003	<0.001

Data are presented as means ± standard deviations. aMCI, amnestic mild cognitive impairment; naMCI, non-amnestic mild cognitive impairment; NC, normal control; MMSE, Mini Mental State Examination; MoCA, Montreal Cognitive Assessment; MIS, Memory Index Score; BNT, Boston Naming Test; Stroop C, Stroop Word Color Test C; CDT, Clock Drawing Test; AVLT, Auditory Verbal Learning Test; VFT, Verbal Fluency Test; SDMT, Symbol Digit Modality Test; DST, Digit Span Test.

**Table 2:** Difference survived FDR correction in cortical thickness among three groups.

	aMCI	naMCI	NC	F	*p* (FDR Corrected)	Post Hoc Groupwise		
						aMCI vs. naMCI	aMCI vs. NC	naMCI vs. NC
Discovery dataset								
rh_G_and_S_cingul_Ant	2.377 ± 0.130	2.294 ± 0.113	2.397 ± 0.139	8.250	<0.001	0.010	1.000	<0.001
rh_G_orbital	2.393 ± 0.116	2.298 ± 0.091	2.395 ± 0.130	8.999	<0.001	0.004	1.000	<0.001
rh_S_orbital_H_Shaped	2.153 ± 0.128	2.061 ± 0.147	2.192 ± 0.166	9.511	<0.001	0.005	0.992	<0.001
rh_S_suborbital	2.125 ± 0.281	1.949 ± 0.212	2.048 ± 0.252	8.046	<0.001	<0.001	0.024	0.109
rh_G_temp_sup_G_T_transv	2.266 ± 0.190	2.188 ± 0.205	2.312 ± 0.222	6.369	0.019	0.005	1.000	0.003
rh_G_front_inf_Opercular	2.329 ± 0.116	2.271 ± 0.148	2.358 ± 0.122	5.930	0.025	0.021	1.000	0.003
Replication dataset								
rh_G_and_S_cingul_Ant	2.564 ± 0.126	2.512 ± 0.124	2.560 ± 0.121	6.340	0.037	0.001	0.086	0.035
rh_G_front_inf_Opercular	2.671 ± 0.129	2.565 ± 0.159	2.655 ± 0.131	7.500	0.037	<0.001	0.164	0.007
rh_G_front_sup	2.845 ± 0.121	2.767 ± 0.137	2.825 ± 0.125	6.635	0.037	0.001	0.027	0.091
rh_G_pariet_inf_Angular	2.567 ± 0.164	2.460 ± 0.157	2.562 ± 0.137	7.058	0.037	<0.001	0.218	0.007
rh_G_front_middle	2.600 ± 0.122	2.519 ± 0.124	2.592 ± 0.121	5.654	0.049	0.001	0.297	0.014

Data are presented as mean ± SD, comparison among groups was performed controlling for age, sex, and education year. F-statistics are reported for the omnibus GLM group effects (df = 2). *p*-values were adjusted for multiple comparisons across regions using the Benjamini–Hochberg FDR procedure. Post hoc pairwise comparisons with LSD adjustment were used to characterize the direction of group differences. G_and_S_cingul_Ant, anterior cingulate gyrus and sulcus; G_front_inf_Opercular, inferior frontal gyrus, opercular part; G_orbital, orbital gyrus; G_temp_sup_G_T_transv, superior temporal gyrus, transverse temporal gyrus; S_orbital_H_Shaped, orbital sulcus, H-shaped; S_suborbital, suborbital sulcus; G_front_middle, middle frontal gyrus; G_front_sup, superior frontal gyrus; G_pariet_inf_Angular, inferior parietal lobule, angular gyrus; rh, right hemisphere; FDR, false discovery rate; LSD, least significant difference.

**Table 3:** Comparisons of subcortical nuclei volumes between the three groups.

	aMCI	naMCI	NC	F	*p* (FDR Corrected)	Post Hoc Groupwise		
						aMCI vs. naMCI	aMCI vs. NC	naMCI vs. NC
Discovery dataset								
Left_Thalamus_Proper	5828.09 ± 530.19	5967.15 ± 727.54	6234.03 ± 677.61	2.882	0.149	–	–	–
Left_Caudate	3310.53 ± 585.76	3291.28 ± 594.38	3331.29 ± 414.61	0.003	0.997	–	–	–
Left_Putamen	4685.58 ± 563.52	4734.38 ± 571.93	4743.28 ± 533.23	0.163	0.915	–	–	–
Left_Pallidum	1726.68 ± 190.66	1814.65 ± 223.93	1815.93 ± 260.62	1.117	0.462	–	–	–
Left_Hippocampus	3512.86 ± 417.48	3723.32 ± 352.91	3782.75 ± 374.33	4.183	0.079	–	–	–
Left_Amygdala	1411.37 ± 249.02	1501.94 ± 239.04	1583.97 ± 252.89	3.460	0.119	–	–	–
Left_Accumbens_area	496.88 ± 75.01	515.40 ± 79.56	534.63 ± 79.38	1.819	0.257	–	–	–
Right_Thalamus_Proper	5820.23 ± 632.01	5923.84 ± 633.40	6173.41 ± 641.51	2.072	0.257	–	–	–
Right_Caudate	3499.36 ± 602.73	3567.63 ± 640.05	3490.45 ± 491.94	0.491	0.722	–	–	–
Right_Putamen	4810.84 ± 645.74	4909.79 ± 592.86	4866.90 ± 546.51	0.481	0.722	–	–	–
Right_Pallidum	1649.38 ± 189.81	1748.69 ± 253.50	1745.26 ± 241.11	1.825	0.257	–	–	–
Right_Hippocampus	3532.62 ± 310.17	3795.44 ± 419.90	3830.16 ± 367.82	7.187	0.014	0.008	<0.001	0.890
Right_Amygdala	1473.45 ± 280.99	1605.48 ± 243.82	1623.56 ± 247.08	2.786	0.149	–	–	–
Right_Accumbens_area	499.20 ± 77.50	512.28 ± 84.02	543.35 ± 82.23	4.388	0.079	–	–	–
Replication dataset								
Left_Thalamus_Proper	6964.91 ± 747.30	7176.90 ± 609.14	7475.82 ± 750.46	5.587	0.028	0.288	0.001	0.095
Left_Caudate	3254.44 ± 477.55	3129.79 ± 314.66	3286.68 ± 416.61	1.387	0.322	–	–	–
Left_Putamen	4591.15 ± 609.08	4511.05 ± 439.91	4702.15 ± 493.93	1.291	0.324	–	–	–
Left_Pallidum	1975.75 ± 262.28	1973.40 ± 221.64	1986.76 ± 204.47	0.075	0.927	–	–	–
Left_Hippocampus	3849.90 ± 322.91	4015.21 ± 279.57	4082.78 ± 368.63	6.569	0.028	0.024	0.001	0.535
Left_Amygdala	1556.15 ± 202.94	1594.91 ± 162.36	1600.60 ± 147.75	0.701	0.535	–	–	–
Left_Accumbens_area	331.37 ± 82.65	358.09 ± 82.98	367.98 ± 86.82	1.489	0.321	–	–	–
Right_Thalamus_Proper	7080.36 ± 781.66	7216.39 ± 755.10	7546.25 ± 783.04	4.207	0.060	–	–	–
Right_Caudate	3254.64 ± 475.56	3162.89 ± 348.42	3345.48 ± 432.53	2.117	0.248	–	–	–
Right_Putamen	4635.91 ± 621.97	4570.72 ± 444.11	4772.59 ± 469.81	1.656	0.302	–	–	–
Right_Pallidum	1930.31 ± 274.04	1899.48 ± 197.38	1987.36 ± 184.86	1.886	0.271	–	–	–
Right_Hippocampus	4057.41 ± 384.20	4285.34 ± 403.75	4302.51 ± 400.01	5.154	0.033	0.013	0.005	0.868
Right_Amygdala	1704.13 ± 212.28	1752.96 ± 197.96	1787.67 ± 163.59	2.631	0.175	–	–	–
Right_Accumbens_area	432.57 ± 79.10	465.78 ± 70.60	471.88 ± 79.94	2.667	0.175	–	–	–

Data are presented as mean ± SD, GLM analysis was performed for comparison among aMCI, naMCI, and NC groups, controlling for age, sex, education year, and total intracranial volume. F-statistics are reported for the omnibus GLM group effects (df = 2). *p*-values were adjusted for multiple comparisons across regions using the Benjamini–Hochberg FDR procedure. Post hoc pairwise comparisons with LSD adjustment were used to characterize the direction of group differences. FDR, false discovery rate; LSD, least significant difference.

## Data Availability

The data presented in this study are available on request from the corresponding author. The data are not publicly available due to privacy and ethical restrictions.

## Supplementary Materials

The following supporting information can be downloaded at: https://www.mdpi.com/article/10.3390/brainsci16010051/s1, Table S1: Number of points with significant difference in pointwise comparison of tract profiles; Table S2: Linear regression results for brain alterations and neuropsychological test scores in MCI; Figure S1: Reanalysis of fiber bundles using more stringent cleaning criteria. Figure S2: Cortical thickness changes among aMCI, naMCI and NC; Figure S3: ComBat harmonization of MRI features; Figure S4: Internal validation performance based on five-fold cross-validation.

## Abbreviations

The following abbreviations are used in this manuscript:

MCI	mild cognitive impairment
aMCI	amnestic mild cognitive impairment
naMCI	non-amnestic mild cognitive impairment
AFQ	automated fiber quantification
ACC	anterior cingulate cortex
IFOF	inferior fronto-occipital fasciculus
AD	Alzheimer’s disease
DTI	diffusion tensor imaging
FA	fractional anisotropy
MRI	magnetic resonance imaging
CDR	Clinical Dementia Rating
MMSE	Mini-Mental State Examination
MoCA	Montreal Cognitive Assessment
MIS	Memory Index Score
BNT	Boston naming test
Stroop C	Stroop color and word test part three
CDT	Clock Drawing Test
AVLT	Auditory Verbal Learning Test
VFT	verbal fluency test
SDMT	symbol digit modality test
DST	digit span test
MD	mean diffusivity
AD	axial diffusivity
RD	radial diffusivity
ATR	anterior thalamic radiation
CST	corticospinal tract
CCing	cingulum cingulate
CHippo	cingulum hippocampus
ILF	inferior longitudinal fasciculus
SLF	superior longitudinal fasciculus
UF	uncinate fasciculus
AF	arcuate fasciculus
CC	corpus callosum
FDR	false discovery rate
IFGop	opercular part of inferior frontal gyrus
