# Supplementary material for: Subtype-Specific Brain Atrophy and White Matter Alterations in Mild Cognitive Impairment
Source: Brain Sci. 2025 Dec 29;16(1):51. doi: 10.3390/brainsci16010051 (PMC12838734; doi:10.3390/brainsci16010051)
Supplement: Supplementary file 1 [file brainsci-16-00051-s001.zip › brainsci-4021604-supplementary.pdf]

Supplementary Tables

Table 1. Number of points with significant difference in pointwise comparison of tract profiles

Discovery dataset									
	Local changes in FA			Local changes in MD			Local changes in AD		
	aMCI vs naMCI	aMCI vs NC	naMCI vs NC	aMCI vs naMCI	aMCI vs NC	naMCI vs NC	aMCI vs naMCI	aMCI vs NC	naMCI vs NC
CC Splenium	–	–	16 (R)	–	–	–	–	–	–
IFOF_R	15 (M)	–	–	–	–	–	–	–	–
UF_L	–	–	–	–	53 (M / F)	–	–	33 (M)	–
**Replication dataset**									
	Local changes in FA			Local changes in AD			Local changes in RD		
	aMCI vs naMCI	aMCI vs NC	naMCI vs NC	aMCI vs naMCI	aMCI vs NC	naMCI vs NC	aMCI vs naMCI	aMCI vs NC	naMCI vs NC
IFOF_R	18 (M)	–	–	–	–	–	–	–	–
SLF_R	44 (M / P)	–	46 (M / P)	–	–	40 (M / P)	38 (M / P)	–	36 (M / P)

NC, normal control; aMCI, amnestic mild cognitive impairment; naMCI, non-amnestic mild cognitive impairment; FA, fractional anisotropy; MD, mean diffusivity; AD, axial diffusivity; RD, radial diffusivity; CC, corpus callosum; IFOF, inferior fronto-occipital fasciculus; UF, uncinated fasciculus; SLF, superior longitudinal fasciculus; L, left; R, right. A, difference in anterior part of the fiber; P, difference in posterior part of the fiber;

M, difference in middle part of the fiber; F, difference in the frontal lobe part of the fiber. FWE corrected with p value < 0.05.

Formatted: Font: Italic

Table 2. Linear regression results for brain alterations and neuropsychological test scores in MCI

Dependent variable	rACC thickness (β, 95% CI, *p*)	rIFGop thickness (β, 95% CI, *p*)	R-HV/TIV (β, 95% CI, *p*)	IFOF_R_FA$_{55-64}$ (β, 95% CI, *p*)
		Discovery dataset		
MMSE	-0.126 (-3.120 – 2.868), 0.934	0.558 (-2.352 – 3.468), 0.704	1.668 (-12.759 – 16.095), 0.819	4.480 (-3.778 – 12.737), 0.284
MoCA	6.570 (3.060 – 10.081), <0.001	5.807 (2.344 – 9.269), 0.001	14.062 (-3.917 – 32.042), 0.124	-25.504 (-34.471 – 16.537), <0.001
MIS	-5.134 (-10.119 – -0.150), 0.044	-3.407 (-8.319 – -1.504), 0.171	28.912 (5.102 – 52.722), 0.018	17.197 (3.512 – 30.882), 0.015
BNT	1.617 (-1.896 – -5.130), 0.363	1.844 (-1.567 – -5.255), 0.286	-4.802 (-21.786 – 12.183), 0.576	2.614 (-7.172 – 12.400), 0.597
Stroop C	-48.377 (-86.383 – -10.372), 0.013	-19.128 (-57.231 – -18.976), 0.321	-2.860 (-192.748 – 187.028), 0.976	179.629 (77.245 – 282.012), 0.001
CDT	1.587 (-0.476 – -3.649), 0.130	0.678 (-1.350 – 2.706), 0.508	-0.580 (-10.654 – 9.493), 0.909	2.006 (-3.781 – 7.794), 0.493
		Replication dataset		
MMSE	2.892 (-1.520 – 7.304), 0.196	0.001 (-2.918 – 2.919), 1.000	-6.108 (-20.586 – 8.369), 0.404	0.820 (-8.286 – 9.926), 0.858
MoCA	7.690 (2.618 – 12.761), 0.003	0.640 (-2.836 – 4.116), 0.716	2.250 (-15.066 – 19.565), 0.797	-19.203 (-30.249 – -8.156), 0.001

Formatted: Font: Italic
Formatted: Font: Italic
Formatted: Font: Italic
Formatted: Font: Italic
Formatted Table
Formatted Table

AVLT	-17.190 (-32.456 – 1.925), 0.028	-14.474 (-24.336 – -4.612), 0.004	55.218 (4.066 – 106.371), 0.035	45.886 (13.630 – 78.141), 0.006
VFT	4.564 (-6.469 – 15.598), 0.413	1.994 (-5.283 – 9.271), 0.588	-12.566 (-49.596 – 24.465), 0.502	-6.843 (-30.872 – 17.185), 0.573
Stroop C	-112.876 (-174.822 – -50.929), <0.001	-14.591 (-57.989 – 28.808), 0.506	-19.207 (-241.552 – 203.139), 0.864	178.954 (46.423 – 311.486), 0.009
SDMT	40.214 (24.283 – 56.145), <0.001	10.923 (-0.957 – 22.803), 0.071	1.829 (-60.158 – 63.816), 0.953	-51.451 (-88.196 – -14.705), 0.007
DST	5.001 (1.050 – 8.952), 0.014	2.039 (-0.612 – 4.691), 0.130	-2.637 (-16.279 – 11.006), 0.702	-4.137 (-12.778 – 4.504), 0.344
CDT	-0.932 (-3.747 – 1.883), 0.513	1.917 (0.105 – 3.730), 0.038	-4.433 (-13.825 – 4.959), 0.351	-2.924 (-8.719 – 2.872), 0.318

Regression coefficients represent unstandardized β estimates with corresponding 95% confidence intervals (CI). P values were adjusted for age, sex and years of education. MMSE, Mini Mental State Examination; MoCA, Montreal Cognitive Assessment; MIS, Memory Index Score; BNT, Boston naming test; Stroop C, Stroop Word Color Test C; CDT, Clock Drawing Test; AVLT, Auditory Verbal Learning Test; VFT, verbal fluency test; SDMT, symbol digit modality test; DST, digit span test; rh, right hemisphere; rACC, right anterior cingulate gyrus and sulcus; rIFGop, right infe-rior frontal gyrus, opercular part; R-HV, right hippocampal volume; TIV, total intracranial volume; IFOF, inferior fronto-occipital fasciculus; FA, fractional anisotropy. *P p < 0.05, **P p < 0.01, ***P p < 0.001.

Formatted: Font: Italic

Supplementary Figures

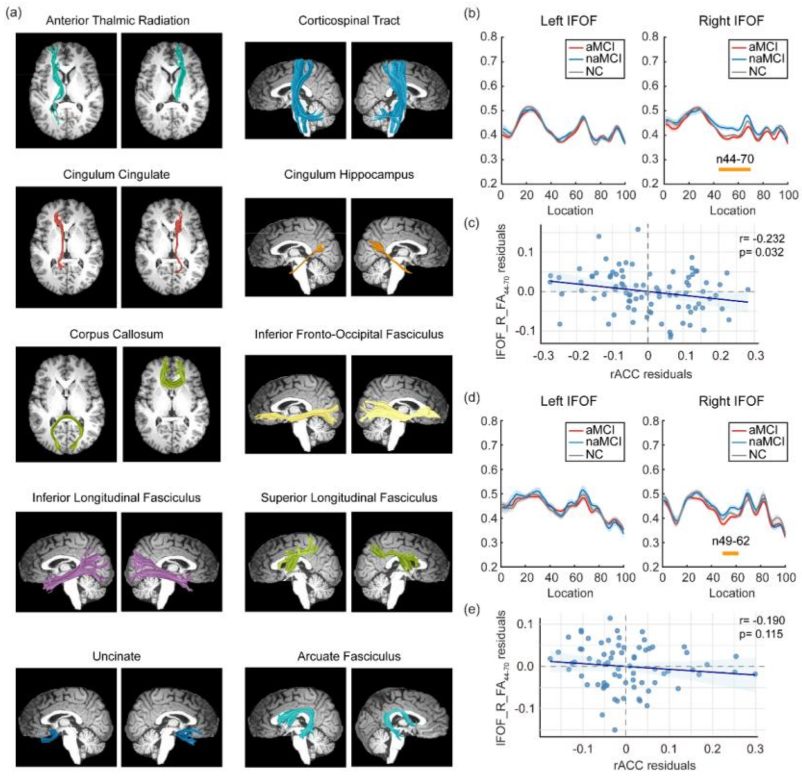

Figure 1. Reanalysis of fiber bundles using more stringent cleaning criteria. (a) 20 fiber bundles retained under more stringent cleaning criteria. Significant results of IFOF FA values from aMCI (red), naMCI (blue) and NC (grey) as means (SD) (solid lines for means and shaded areas for SDs) are plotted, in the discovery dataset (b) and the replication dataset (d). The yellow color bar under the profiles indicate the regions of significant difference for aMCI vs. naMCI. The scatter plot displays the partial correlation between the residuals of right IFOF local values and residuals of right ACC thickness after regressing out the effects of sex, age and years of education. The solid line indicates the fitted linear regression line, with the shaded area representing the 95%

confidence interval. Each point represents an individual participant, * $p < 0.05$. IFOF, inferior fronto-occipital fasciculus; FA, fractional anisotropy; ACC, anterior cingulate cortex.

Formatted: Font: Italic

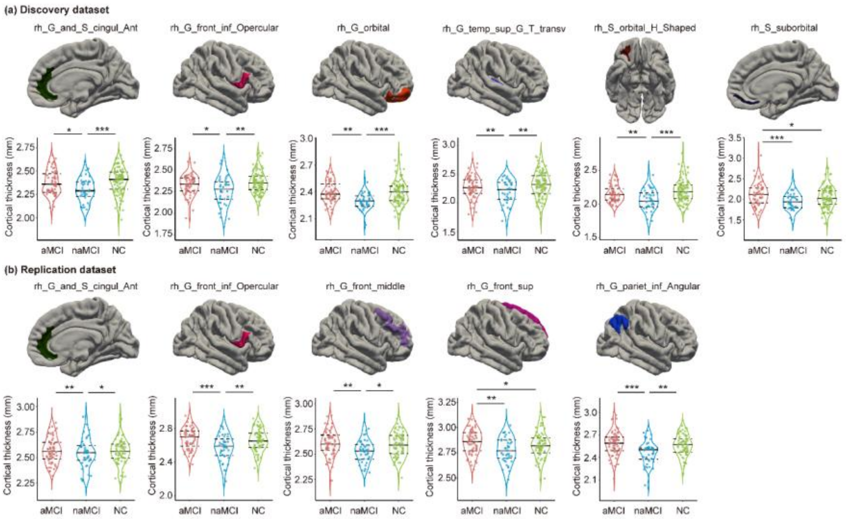

Figure 2. Cortical thickness changes among aMCI, naMCI and NC. Cortical regions with significant difference are shown on the surface of the brain. The violin plots show the difference in cortical thickness among three groups. G_and_S_cingul_Ant, anterior cingulate gyrus and sulcus; G_front_inf_Opercular, inferior frontal gyrus, opercular part; G_orbital, orbital gyrus; G_temp_sup_G_T_transv, superior temporal gyrus, transverse temporal gyrus; S_orbital_H_Shaped, orbital sulcus, H-shaped; S_suborbital, suborbital sulcus; G_front_middle, middle frontal gyrus; G_front_sup, superior frontal gyrus; G_pariet_inf_Angular, inferior parietal lobule, angular gyrus; rh, right hemisphere. Comparison was performed controlling for age, sex and education year, *p < 0.05, **p < 0.01, ***p < 0.001.

Formatted: Font: Italic

Formatted: Font: Italic

Formatted: Font: Italic

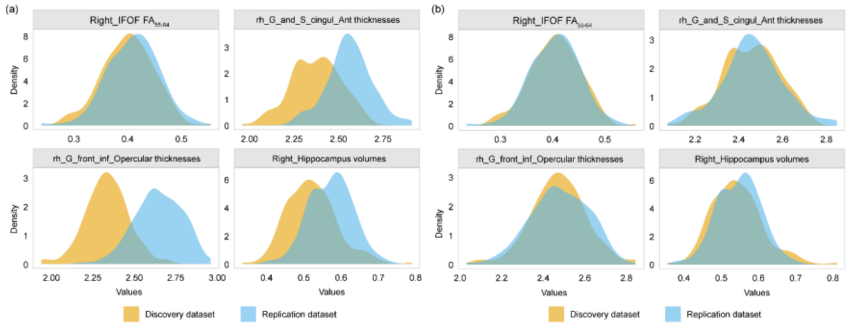

Figure 3. ComBat harmonization of MRI features. Cortical thicknesses of right anterior cingulate and opercular part of inferior frontal gyrus, right hippocampus volume, and local FA value of the right IFOF were harmonized by ComBat. For each feature, probability density function (%) calculated from the discovery dataset and the replication dataset is plotted here before (a) and after (b) ComBat realignment.

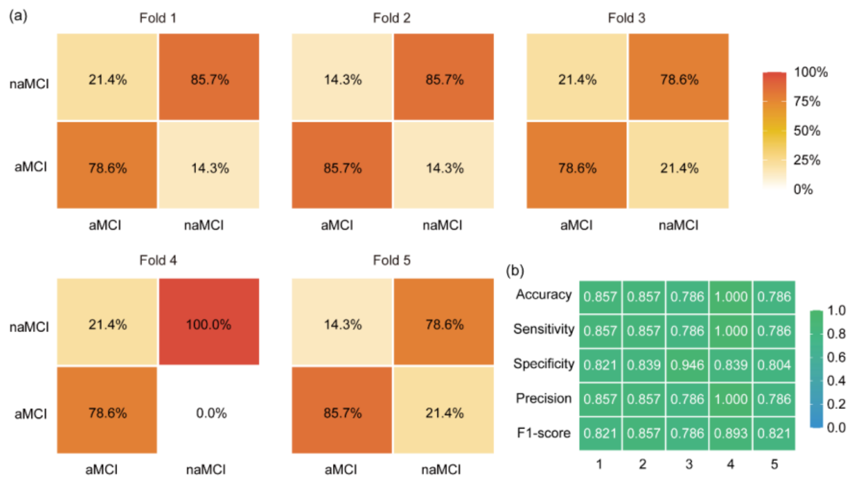

Figure 4. Internal validation performance based on five-fold cross-validation. (a) Confusion matrices for each validation fold, displayed as percentages normalized within each true class. Values represent the proportion of subjects classified as aMCI or naMCI for a given true category, with each row summing to 100%. (b) Heatmap summarizing key performance metrics, including accuracy, sensitivity (recall), specificity, precision, and F1 score, for each validation fold. Color intensity reflects metric values ranging from 0 to 1, with higher values indicating better performance.
